# Practical implementation of a q4–q16 aflibercept treat-and-extend pathway for the treatment of neovascular age-related macular degeneration: Updated guidance from a UK expert panel

**DOI:** 10.1038/s41433-022-02264-3

**Published:** 2022-10-07

**Authors:** Clare Bailey, Peter Cackett, Ajay Kotagiri, Sajjad Mahmood, Evangelos Minos, Nirodhini Narendran, Ashish Patwardhan, Dawn A. Sim, Peter Morgan-Warren, Carolyn O’Neil, Katie Straw

**Affiliations:** 1 University Hospitals Bristol and Weston NHS Foundation Trust, Bristol, UK; 2 Princess Alexandra Eye Pavilion, NHS Lothian, Edinburgh, UK; 3 South Tyneside and Sunderland NHS Foundation Trust, Sunderland, UK; 4 University of Manchester, Manchester, UK; 5 North West Anglia NHS Foundation Trust, Peterborough, UK; 6 The Royal Wolverhampton NHS Trust, Wolverhampton, UK; 7 Royal Cornwall Hospitals NHS Trust, Cornwall, UK; 8 Moorfields Eye Hospital NHS Foundation Trust, London, UK; 9 Bayer plc, Reading, UK

**Keywords:** Medical research, Health care

## Abstract

**Objectives:**

This report, based on guidance from a panel of UK retina specialists, introduces a revised intravitreal aflibercept (IVT-AFL) treat-and-extend (T&E) pathway for the treatment of neovascular age-related macular degeneration (nAMD). The T&E pathway incorporates the updated IVT-AFL label (April 2021) allowing flexible treatment intervals of 4 weeks to 16 weeks, after three initiation doses and a further dose after 8 weeks. Practical guidance is provided on the clinical implementation of the revised pathway, with the aim of supporting clinical decision-making to benefit patients and addressing capacity issues in nAMD services.

**Methods:**

Three structured round-table meetings of UK retina specialists were held online on 19 May, 16 June and 13 October 2021. These meetings were organised and funded by Bayer.

**Results:**

The authors revised the previously published consensus pathway to reflect the changes to the IVT-AFL label and developed guidelines for the implementation of the pathway in UK clinical practice. The guidelines include topics such as recommendations for extending patients with 2- or 4-week adjustments, extending patients to 16-week treatment intervals, managing fellow eye involvement, and reducing treatment intervals for patients with particularly active disease.

**Conclusions:**

The revised IVT-AFL T&E nAMD pathway offers guidance to clinicians seeking to increase the dosing flexibility of IVT-AFL, with 4- to 16-week treatment intervals, in line with the updated IVT-AFL label, to meet the continually evolving demands of nAMD service provision.

## Introduction

Anti–vascular endothelial growth factor (VEGF) regimens for the treatment of neovascular age-related macular degeneration (nAMD) have evolved over time. Evidence from clinical trials and real-world studies demonstrate three main intravitreal anti-VEGF treatment regimens: proactive fixed regimens, reactive *pro re nata* (PRN) regimens and proactive treat-and-extend (T&E) regimens, with T&E regimens having become the mainstay for the treatment of nAMD in many countries [1, 2].

Fixed treatment regimens provide predictability and can simplify decision-making, but may result in over- or undertreatment if the treatment interval is not optimal [2]. PRN regimens have been associated with poorer visual outcomes in clinical practice than in randomised controlled trials because of challenges such as undertreatment and lack of patient adherence and compliance to the retreatment criteria used by the treating clinician [2, 3]. Additionally, patients receiving PRN regimens require monthly monitoring to determine the need for treatment, adding to capacity burden [2]. The fear of negative examination results and the risk of disease recurrence can lead to increased patient anxiety with PRN regimens [2, 4]. AURA, an international retrospective study that assessed the effectiveness of anti-VEGF treatment regimens in patients with nAMD, concluded that fewer injections are administered in clinical practice than in clinical trials and that undertreatment is often associated with poor visual outcomes over time [5]. T&E regimens offer a proactive, personalised treatment approach and are associated with a reduced burden for patients and healthcare systems when compared with PRN regimens while often optimising visual outcomes [3]. Furthermore, the predictability of T&E regimens may help to manage clinic flows, aiding capacity planning [2, 6, 7].

Key data supporting intravitreal aflibercept (IVT-AFL) T&E for the treatment of nAMD are provided by the ALTAIR and ARIES studies [6, 8]. In Year 1 of the ALTAIR study, all patients received three initial monthly doses of IVT-AFL and a further dose after 8 weeks, prior to 1:1 randomisation at Week 16 to either 2- or 4-week treatment interval adjustments. Treatment intervals ranged from 8 weeks to 16 weeks throughout the study. Good functional and anatomical outcomes were achieved up to Week 96 in both groups receiving IVT-AFL T&E in Year 1 [8]. Similarly, the ARIES study demonstrated that good visual outcomes were maintained to Week 104 in both the early-start T&E (T&E from Year 1 with 2-week adjustments) and the late-start T&E (fixed 8-week regimen in Year 1, followed by 2-week T&E adjustments in Year 2) groups. Both groups received three initial monthly IVT-AFL doses and a further dose after 8 weeks, prior to 1:1 randomisation at Week 16 [6]. The minimum treatment interval for both groups was 8 weeks and the maximum treatment interval was 16 weeks, although patients could be treated with a minimum treatment interval of 4 weeks based on clinical judgement [9]. Following the ALTAIR study, IVT-AFL received a label variation in Europe in 2018, allowing T&E in Year 1 after three initial monthly doses and an additional dose after 8 weeks, with a minimum treatment interval of 8 weeks [10].

Although many UK NHS medical retina services have expanded to meet the growing demand for anti-VEGF injections, it has been difficult, and nAMD case numbers are predicted to increase by 59–64% from 2015 to 2035 [11–13]. Additionally, the COVID-19 pandemic has posed considerable challenges to treatment delivery. Multiple factors, including staff absences, requirements to reduce hospital attendances, and patient-initiated cancellations due to fear of COVID-19 infection, have led to treatment disruption [14, 15]. A UK study showed that 36.6% of patients with nAMD, who received at least one anti-VEGF injection between 1 January 2020 and 23 March 2020, experienced a delay in requested review of 8 weeks or more. [14]. During the early 2020 lockdown period, the Royal College of Ophthalmologists recommended maintaining patients on fixed 8-week dosing with anti-VEGF agents, with minimal monitoring [16].

Despite the availability of data and the unprecedented pressures on services, treatment intervals of up to 16 weeks with IVT-AFL are not always used in clinical practice. This may be due to low awareness of the data supporting extension to 16-week intervals, and many clinicians may feel that the achievement of stable disease at 12-week intervals for 1 year is sufficient to discontinue treatment and commence monitoring.

In April 2021, the IVT-AFL product licence in Europe for the treatment of patients with nAMD was extended to include the option of dosing at 4-week intervals in Year 1 of treatment, after the initiation phase (often referred to as the loading phase) of three initial monthly doses and an additional dose after 8 weeks [17]. This update offers greater flexibility in the dosing of IVT-AFL as part of a proactive T&E regimen in Year 1. There is now a need to update existing T&E protocols to reflect this licence extension and the data from ARIES further supporting the use of IVT-AFL with up to 16-week treatment intervals. This publication serves as an update to the Ross et al. 2020 consensus, which provided recommendations for the implementation of a T&E pathway prior to the 2021 product licence extension [18].

The objectives of this publication are to introduce the revised T&E pathway and provide expert-led practical guidance on its clinical implementation. The publication will address how the revised pathway may help to support clinical decision-making and alleviate the capacity and resource issues that continue to strain nAMD services.

## Methods

Three structured round-table meetings of UK retina specialists were held online on 19 May, 16 June and 13 October 2021. These meetings were organised and funded by Bayer and were set up to develop a revised T&E pathway that reflects the updated IVT-AFL licence in nAMD and to explore the practical guidance required for its implementation within a standard UK clinic.

Development of the T&E pathway was supported by a pre-meeting survey of current clinical practice and use of IVT-AFL. During the online meetings, further data on the use of T&E regimens were reviewed and the panel discussed predefined questions developed by Bayer to support pathway generation.

## Guidance from a UK expert panel

### Overview

**Fig. 1:**
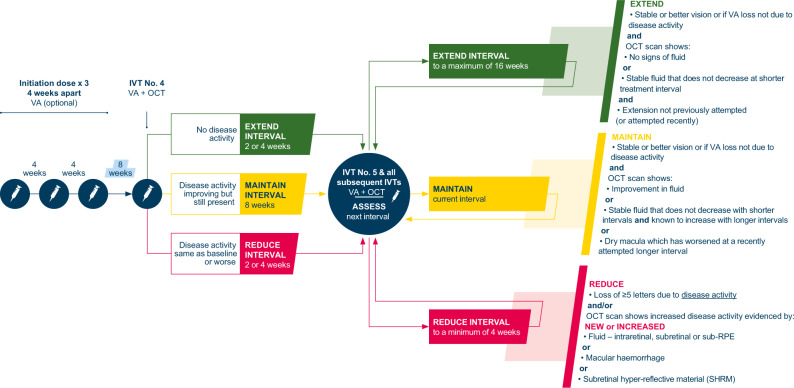
The revised IVT-AFL T&E pathway for the treatment of patients with nAMD. IVT Intravitreal injection, IVT-AFL Intravitreal aflibercept, nAMD Neovascular age-related macular degeneration, OCT Optical coherence tomography, RPE Retinal pigment epithelium, T&E Treat-and-extend, VA Visual acuity.

The revised IVT-AFL T&E pathway is presented in Fig. 1.

The initiation phase consists of three consecutive IVT-AFL doses at 4-week intervals. Visual acuity (VA) assessment is optional during the initiation phase and at the discretion of the treating clinician.

The fourth IVT-AFL dose is given 8 weeks after the third initiation dose. VA assessment and optical coherence tomography (OCT) imaging should be performed at this visit, and at all subsequent intravitreal injection (IVI) visits, to determine the succeeding treatment interval.

After the fourth IVT-AFL dose, the treatment interval is either extended, maintained or reduced; criteria for each decision are shown in Fig. 1.

### Extending with 2- or 4-week adjustments

The licensed posology of IVT-AFL allows injection intervals to be increased by 2 or 4 weeks to maintain stable vision and/or anatomical outcomes [17].

In the ARIES study, both patients randomised to early-start T&E and to late-start T&E showed improved visual outcomes from baseline to Week 104. The mean change in best corrected VA from Week 16 to Week 104 with the early-start T&E regimen was non-inferior to that with the late-start T&E regimen. The mean number of injections was comparable between groups (12.0 versus 13.0 injections for early-start T&E and late-start T&E, respectively) [6].

The ALTAIR study demonstrated that visual outcomes were maintained in patients receiving IVT-AFL with either 2- or 4- week adjustments. From baseline to Week 96, mean gains in best corrected VA were 7.6 and 6.1 Early Treatment Diabetic Retinopathy Study letters for the 2- and 4-week extension groups, respectively. Furthermore, the mean number of injections was the same for both groups over 96 weeks (10.4 injections). The ALTAIR study also showed that patients extended with 2-week adjustments were more likely than patients receiving 4-week adjustments to maintain 16-week treatment intervals to Week 96. Up to 96.3% of patients in the 2-week extension group who were extended to 16-week treatment intervals during the study were maintained on 16-week intervals to Week 96, compared with 77.6% of patients in the 4-week extension group [8].

The revised T&E pathway shown in Fig. 1 allows for treatment interval extensions to be controlled but provides flexibility to extend patients quickly if they are responding well.

Recommendation: We recommend that most patients should be extended with 2-week adjustments; however, 4-week adjustments can be considered for patients with stable or improved VA and a completely dry macula after the initiation phase. The response to the three initiation doses can be used to determine whether 2-week or 4-week adjustments can be made after the fourth dose has been received (8 weeks after the third initiation dose). A qualitative assessment of OCT can determine these adjustments along with clinical judgement.

### Extending to 16-week treatment intervals

Treatment intervals with IVT-AFL for nAMD can be extended incrementally to a maximum of 16 weeks [17]. By Week 96 of the ALTAIR study, 41.5% and 46.3% of patients reached the maximum last treatment interval of 16 weeks with 2- and 4-week adjustments, respectively [8]. Furthermore, in the ARIES study, 30.2% of patients in the early-start T&E group and 26.9% of patients in the late-start T&E group reached a last injection interval of ≥ 16 weeks up to Week 104 (intervals of greater than 16 weeks were considered to be minor deviations and were included in the per-protocol set) [6].

The effect of treatment delays with anti-VEGF agents due to the COVID-19 pandemic was explored by Stone et al. Patients with nAMD, diabetic macular oedema or retinal vein occlusion who received at least one anti-VEGF injection between 1 January 2020 and 23 March 2020 experienced a mean delay in requested review of 13.1 weeks. Despite this delay, 26.7% of eyes with nAMD remained dry and 61.9% of patients with nAMD maintained their VA (within 5 letters of their baseline) [14]. Data from this study suggest that more ambitious treatment extension intervals could be attempted in some patients.

Recommendation: The clinical trial data support extending suitable patients to 16-week treatment intervals. The guidance in this report may be used when considering extension.

### Reducing treatment intervals

Alongside the treatment of active disease, prevention of disease recurrence and/or worsening also defines the success of anti-VEGF agents [2]. In our UK practices, 10–12% of patients require injections at intervals of less than 8 weeks. Additionally, data from ARIES demonstrated that 5.7–7.7% of patients were maintained with a last treatment interval of less than 8 weeks at Week 104 [6].

The recommended criteria for reducing treatment intervals are a loss of ≥5 letters due to disease activity, or OCT imaging showing increased disease activity, evidenced by new or increased fluid (intraretinal, subretinal or subretinal pigment epithelium), macular haemorrhage or subretinal hyperreflective material.

Recommendation: The revised T&E pathway (Fig. 1) allows for shortening of the treatment interval to 4 or 6 weeks in Year 1 for patients with particularly active disease. To prevent frequent disease recurrences, it is advised that clinicians decide how many episodes of disease recurrence to tolerate, before deciding not to extend to that treatment interval again. Additionally, we recommend that, after a failed treatment extension, treatment should recommence at the shorter interval received prior to the failed extension. Treatment interval extensions following disease recurrence should be determined by the treating clinician. Shortening treatment intervals may be temporary for some patients, because disease activity changes over time; therefore, treatment interval extensions should not be entirely dismissed based on previous unsuccessful attempts. Patient factors such as vision in the fellow eye are important to consider prior to re-attempting interval extensions.

After one failed extension, we advise that fixed dosing at the last effective treatment interval should recommence for 4–6 months prior to re-attempting extension, and for 1 year after two or more failed extensions, depending on recurrence severity.

### Performing injection-only visits for stable patients on planned fixed treatment intervals

In accordance with the licensed IVT-AFL posology, there is no requirement for monitoring between injections, and the frequency of monitoring is to be determined by the treating clinician [17]. The Royal College of Ophthalmologists advises clinicians to be aware of the increased risk of long-term raised intraocular pressure in patients receiving regular IVI, and to monitor intraocular pressure according to clinical concern and risk [19]. In the absence of specific concerns or risk factors, a minimum frequency of annual intraocular pressure assessments may be appropriate.

Recommendation: For services constrained by capacity issues, we recommend that injection-only visits without diagnostics be considered for patients who are stable at planned treatment intervals. Injection-only visits can aid capacity planning; however, local policies should be followed because these visits are dependent on the set-up of individual clinics and clinical assessment by the treating clinician, including consideration of possible second eye involvement.

The number of permitted injection-only visits should be determined by the patient’s current injection interval. The duration that the interval should be maintained prior to commencing injection-only visits is at the discretion of the treating clinician and is dependent on factors such as the number of previous failed treatment extensions and how recently interval extension has been attempted. For patients receiving IVI at a fixed treatment interval of 4 weeks, a maximum of three injection-only visits are recommended before performing diagnostics. For patients at a fixed treatment interval of 6 weeks, a maximum of two injection-only visits before performing diagnostics is preferred. For patients with a treatment interval of 8 weeks, one injection-only visit is recommended before performing diagnostics, whereas diagnostics are recommended at each clinic visit for patients at intervals over 8 weeks. Where suitable, outsourcing (or insourcing) imaging hubs managed by hospital medical retina teams, could potentially help with capacity restraints.

### Managing fellow eye involvement

Patients with nAMD in one eye have a high risk of developing nAMD in the fellow eye. In a study performed by Moorfields Eye Hospital, UK, the fellow eye involvement rate was 32% at Year 2, with a median interval to fellow eye involvement of 71 weeks [20].

A high proportion of fellow eyes with nAMD are asymptomatic at diagnosis, with the disease detected by OCT scanning or fundal examination during follow-up appointments of first-treated eyes. This highlights the importance of regular monitoring of fellow eyes, ensuring early detection to prevent treatment delay, which is crucial for maintaining vision and quality of life [21, 22].

Recommendation: Unless the second eye is untreatable, we recommend monitoring both eyes with OCT. For certain patients, clinical circumstances may require OCT imaging even if the fellow eye is untreatable (for example, to allow patients to understand the mechanisms behind their deteriorating vision).

To promote efficient implementation of services, increased patient convenience and improved patient compliance, synchronisation of treatment and monitoring of the more active eye should be considered where possible.

For unaffected fellow eyes, OCT imaging should be performed every 12–16 weeks, during diagnostic visits for the affected eye. Additionally, patients (especially those with long treatment intervals) should be told to remain vigilant for evidence of disease activity and to report any symptoms as soon as they occur.

### Investigating for differential diagnoses

Polypoidal choroidal vasculopathy is a subtype of nAMD. Indocyanine green imaging can be used to visualise the vasculature of the choroid and thereby add value when differentiating between polypoidal choroidal vasculopathy and typical nAMD [23, 24].

Recommendation: We recommend considering fluorescein angiography and/or indocyanine green imaging to assess for polypoidal choroidal vasculopathy or other pathologies if the patient has a poor response to treatment, or there is no change or worsening on OCT imaging at Visit 4 (after the three initiation doses and an interval of 8 weeks). If disease activity remains unchanged by Visit 4, the treatment interval should be reduced as per the revised T&E pathway (Fig. 1) and the treating clinician should monitor the responsiveness to the decreased treatment interval prior to investigating for alternative diagnoses.

### Discontinuing treatment

There are limited data on the risk of disease recurrence after treatment cessation and on post-treatment recurrence outcomes. Disease recurrence rates in nAMD are high; a 2021 study showed that 52.9% of patients who were followed-up for 12 months after treatment cessation showed evidence of disease recurrence, with a mean time to recurrence after the last injection of 6.7 months [25]. Furthermore, 40.7% of patients with recurrent disease did not show symptoms of visual loss or metamorphopsia during follow-up [25].

Recommendation: Treatment cessation should be discussed with long-term stable patients who have received at least three injections at a treatment interval of 16 weeks with no disease activity. Some UK clinics adopt a ‘monitor-and-extend’ regimen to monitor patients who have stopped treatment; this type of monitoring is most suitable for a virtual clinic approach. A monitor-and-extend regimen initially involves monitoring patients with VA and OCT assessments 4 weeks after the last missed dose. After this, if nAMD remains inactive, monitoring intervals can be incrementally increased at the discretion of the treating clinician. Even though long-term follow-up with OCT would be ideal, capacity constraints mean that in many units, patients who have been monitored for 2 years without disease recurrence are discharged with self-monitoring and annual sight tests with their local optometrist. However, some clinics discharge low-risk patients (with the exclusion of patients with only one treatable eye and vulnerable adults) after discussion, before completion of 2 years of monitoring.

To capture patients who experience disease recurrence, we recommend that clinics have a fast-track referral system in place for patients who are monitored by optometrists or discharged.

Treatment might not only be discontinued in those with stable disease who perform well. A subset of patients may wish to prematurely end treatment for reasons such as treatment fatigue or lack of perceived efficacy. Nursing staff and clinicians may also identify patients who are no longer benefiting from treatment at injection clinics. We recommend that clinics establish internal policies for periodic reviews with supervising consultants to discuss treatment cessation.

Prior to discharge, we recommend that patients be reviewed face-to-face by a clinician to confirm discharge eligibility and exclude any ocular co-pathology requiring treatment.

### Managing disease relapse after treatment cessation

The severity of a disease relapse should be determined by the treating clinician.

Recommendation: We recommend that, for stable patients who have been treatment-free (defined as a patient extended to at least three 16-week intervals and a decision made to stop treatment) for 0–2 years presenting with disease relapse, a decision should be made on whether to restart the pathway or determine injection frequency based on disease severity and previous response to treatment.

## Guidance on the implementation of the revised pathway

Further guidance on the implementation of the revised T&E pathway is shown in Table 1. The aim of this guidance is to provide support to UK clinics looking to optimise their services following the 2021 IVT-AFL product licence extension. The additional guidance includes considerations for services setting up a T&E regimen, appointment scheduling to optimise clinic capacity, and patient support.

**Table 1:** Guidance for implementing the revised IVT-AFL T&E pathway.

**Initial set-up**
Gain approval from all team members, including the business manager, lead clinicians and administrative staff, and hold a meeting to explain the protocol.
Ensure that there is a coordinator and dedicated administration team to run the service.
**Planning of interval extensions**
Use the response to the initiation doses to gauge interval extension lengths.
Consider 2-week treatment interval extensions for most patients; however, consider 4-week treatment extensions for patients with a completely dry macula after the initiation phase.
If disease recurs after extension, consider keeping patients on a shorter interval for a minimum period of time (e.g., 6 months after the first recurrence and 12 months after subsequent recurrences).
Decide how many extensions with disease recurrence will be tolerated before deciding not to extend to that interval again.
Ensure that posters of the T&E pathway are displayed in clinic rooms and that T&E guidelines are available on the intranet.
**Appointment scheduling**
Optimise clinic capacity to deliver injections on time and increase adherence by enabling patients to book their next appointment during a visit.
Consider injection-only visits without diagnostics for patients who are stable at a set interval.
Create a robust method for rebooking patients who do not attend their appointment. Decide if extension should be from the original appointment date or the rescheduled appointment date when the patient next attends the clinic.
**Monitoring and discharge**
Unless the second eye is untreatable, monitor both eyes with OCT and plan for managing second eye involvement.
Plan a discharge policy to increase capacity. For example, discharge patients to OCT monitoring in virtual clinics after three 16-week intervals and discharge patients from the macula clinic if they have end-stage nAMD.
Advise members of the multidisciplinary team to discuss patients who are non-responsive to treatment with a consultant.
**Patient support**
Ensure that patient information leaflets (including information on the concept of T&E and recognising signs of disease recurrence) are available in clinics. Distribute these leaflets at the first outpatient appointment at the time of diagnosis.
Ensure that patients are regularly assessed for eligibility for sight impairment registration and that prompt referrals to low-vision clinics are made where appropriate.
Ensure that patients have an emergency contact number to be used in the event of acute visual deterioration (including for out-of-hours) and an administrative contact for patient-initiated follow-up.

*IVT-AFL* intravitreal aflibercept, *nAMD* neovascular age-related macular degeneration, *OCT* optical coherence tomography, *T&E* treat-and-extend.

## Closing comments

It is predicted that the nAMD disease burden will continue to rise over the next decade, leading to an increased demand for IVI and added pressures for services across the UK [11, 12].

There are limited studies published showing real-world outcomes of anti-VEGF treatment over many years. A retrospective review, published in 2021, demonstrated that maintaining anti-VEGF treatment for nAMD sustains vision over a 10-year period, with 63.3% of eyes losing ≤ 15 letters of VA after 10 years of treatment [26]. However, there remains uncertainty in the response to anti-VEGF treatment for nAMD after 10 years [26]. Further real-world data are required to support additional developments in nAMD management, including long-term outcomes of different treatment regimens, the frequency of disease recurrence after treatment cessation, and the long-term visual outcomes after these recurrences. Additional data will help to support medical retina specialists with the decision-making around treatment cessation and discharge, which could address capacity issues.

Additionally, it is important to note that increased reliance on retinal imaging in the management of nAMD does not replace a holistic approach to eye care. Significant ocular comorbidities, such as cataract and glaucoma, can be overlooked in practice when the major focus is on nAMD and treatment pathways; therefore, it is important that patients are encouraged to seek help should they experience any symptoms or require urgent advice.

Moreover, it is important for patients to understand that treatment for nAMD is a long-term undertaking; this should be made clear at treatment initiation and as therapy continues. Patients who are considering anti-VEGF discontinuation because of treatment fatigue should be reminded of this need for long-term therapy and the potential for disease recurrence [25].

To offer increased patient support and to reduce the number of follow-up appointments, it is also recommended that patients have access to an optometrist trained in low visual aid and an eye clinic liaison officer, including during discharge consultations [24, 27].

The revised IVT-AFL T&E pathway for the treatment of nAMD outlined in this publication is based on our clinical experience as UK medical retina specialists. The revised pathway provides clear guidance and demonstrates how the updated IVT-AFL label can be followed in clinical practice. The aim of implementing the revised pathway is to help services to efficiently address and alleviate capacity issues while improving patient experience and clinical care and treatment outcomes.

## Summary

### What was known before

- T&E regimens have become the mainstay for the treatment of nAMD in many countries, which may decrease the treatment burden for patients and healthcare systems while maintaining good visual outcomes.
- In April 2021, the product licence in Europe for IVT-AFL was extended for the treatment of patients with nAMD, allowing the option of dosing at 4-week intervals in Year 1 of treatment, after the initiation phase of three monthly doses and a further dose after 8 weeks.

### What this study adds

- A revised proactive T&E pathway for the treatment of nAMD with IVT-AFL, allowing treatment intervals of 4 weeks to 16 weeks, after three initial monthly doses and a further dose after 8 weeks, with guidance for treatment extension, reduction and maintenance.
- Expert-led practical recommendations from UK retina specialists on the clinical implementation of the extended IVT-AFL label, addressing how the revised T&E pathway may help to support clinical decision-making and address the capacity and resource issues that strain nAMD services.

## References

[1] Mitchell P, Liew G, Gopinath B, Wong TY (2018). Age-related macular degeneration. Lancet.

[2] Lanzetta P, Loewenstein A, Vision Academy Steering Committee. (2017). Fundamental principles of an anti-VEGF treatment regimen: optimal application of intravitreal anti-vascular endothelial growth factor therapy of macular diseases. Graefes Arch Clin Exp Ophthalmol.

[3] Daien V, Finger RP, Talks JS, Mitchell P, Wong TY, Sakamoto T (2021). Evolution of treatment paradigms in neovascular age-related macular degeneration: a review of real-world evidence. Br J Ophthalmol.

[4] Droege KM, Muether PS, Hermann MM, Caramoy A, Viebahn U, Kirchhof B (2013). Adherence to ranibizumab treatment for neovascular age-related macular degeneration in real life. Graefes Arch Clin Exp Ophthalmol.

[5] Holz FG, Tadayoni R, Beatty S, Berger A, Cereda MG, Cortez R (2015). Multi-country real-life experience of anti-vascular endothelial growth factor therapy for wet age-related macular degeneration. Br J Ophthalmol.

[6] Mitchell P, Holz FG, Hykin P, Midena E, Souied E, Allmeier H (2021). Efficacy and safety of intravitreal aflibercept using a treat-and-extend regimen for neovascular age-related macular degeneration: the ARIES study: a randomized clinical trial. Retina.

[7] Amoaku W, Bailey C, Downey L, Gale RP, Ghanchi F, Hamilton R (2020). Providing a safe and effective intravitreal treatment service: strategies for service delivery. Clin Ophthalmol.

[8] Ohji M, Takahashi K, Okada AA, Kobayashi M, Matsuda Y, Terano Y (2020). Efficacy and safety of intravitreal aflibercept treat-and-extend regimens in exudative age-related macular degeneration: 52- and 96-week findings from ALTAIR; a randomized controlled trial. Adv Ther.

[9] Bayer data on file (EYL028). ARIES: Integrated Clinical Study Protocol. No. BAY86-5321/17508. 2019.

[10] European Medicines Agency. Eylea: EPAR – Procedural steps taken and scientific information after the authorisation. 2021. https://www.ema.europa.eu/en/documents/procedural-steps-after/eylea-epar-procedural-steps-taken-scientific-information-after-authorisation_en.pdf.

[11] Gale RP, Mahmood S, Devonport H, Patel PJ, Ross AH, Walters G (2019). Action on neovascular age-related macular degeneration (nAMD): recommendations for management and service provision in the UK hospital eye service. Eye (Lond).

[12] Buchan JC, Norman P, Shickle D, Cassels-Brown A, MacEwen C (2019). Failing to plan and planning to fail. Can we predict the future growth of demand on UK Eye Care Services?. Eye (Lond).

[13] The Royal College of Ophthalmologists. The Way Forward. Age-Related Macular Degeneration and Diabetic Retinopathy. 2017. https://www.rcophth.ac.uk/wp-content/uploads/2015/10/RCOphth-The-Way-Forward-AMD-300117.pdf.

[14] Stone LG, Grinton ME, Talks JS (2021). Delayed follow-up of medical retina patients due to COVID-19: impact on disease activity and visual acuity. Graefes Arch Clin Exp Ophthalmol.

[15] Appleby J (2021). NHS sickness absence during the covid-19 pandemic. BMJ.

[16] The Royal College of Ophthalmologists. Guidance on restarting Medical Retina Services. 2020. https://www.rcophth.ac.uk/wp-content/uploads/2021/01/Guidance-on-restarting-Medial-Retina-Services-1.pdf.

[17] Bayer AG. Eylea 40mg/mL solution for injection in a vial – summary of product characteristics. 2021.

[18] Ross AH, Downey L, Devonport H, Gale RP, Kotagiri A, Mahmood S (2020). Recommendations by a UK expert panel on an aflibercept treat-and-extend pathway for the treatment of neovascular age-related macular degeneration. Eye (Lond).

[19] The Royal College of Ophthalmologists. Intravitreal injection therapy. Ophthalmic Service Guidance report. 2018. https://www.rcophth.ac.uk/wp-content/uploads/2022/02/Intravitreal-Injection-Therapy-August-2018-1.pdf.

[20] Fasler K, Fu DJ, Moraes G, Wagner S, Gokhale E, Kortuem K (2020). Moorfields AMD database report 2: fellow eye involvement with neovascular age-related macular degeneration. Br J Ophthalmol.

[21] Chew JK, Zhu M, Broadhead GK, Luo K, Hong T, Chang AA (2017). Bilateral neovascular age-related macular degeneration: comparisons between first and second eyes. Ophthalmologica.

[22] Zarranz-Ventura J, Liew G, Johnston RL, Xing W, Akerele T, McKibbin M (2014). The neovascular age-related macular degeneration database: report 2: incidence, management, and visual outcomes of second treated eyes. Ophthalmology.

[23] Palkar AH, Khetan V (2019). Polypoidal choroidal vasculopathy: an update on current management and review of literature. Taiwan J Ophthalmol.

[24] The Royal College of Ophthalmologists. Age Related Macular Degeneration Services Commissioning Guidance. 2021. https://www.rcophth.ac.uk/wp-content/uploads/2021/07/AMD-Commissioning-Guidance-Full-June-2021.pdf.

[25] Aslanis S, Amrén U, Lindberg C, Epstein D (2021). Recurrent neovascular age-related macular degeneration after discontinuation of vascular endothelial growth factor inhibitors managed in a treat-and-extend regimen. Ophthalmol Retin.

[26] Cheema MR, DaCosta J, Talks J (2021). Ten-year real-world outcomes of anti-vascular endothelial growth factor therapy in neovascular age-related macular degeneration. Clin Ophthalmol.

[27] Young SL, Ng N, Yap NJ, Hussain Z, Cackett PD. A novel multidisciplinary approach to the management of end-stage macular disease. Br J Vis Impair. 2021. 10.1177/02646196211032694.

[28] Battisti WP, Wager E, Baltzer L, Bridges D, Cairns A, Carswell CI (2015). Good Publication Practice for Communicating Company-Sponsored Medical Research: GPP3. Ann Intern Med.

